# Heterozygosity for a Hypomorphic Polβ Mutation Reduces the Expansion Frequency in a Mouse Model of the Fragile X-Related Disorders

**DOI:** 10.1371/journal.pgen.1005181

**Published:** 2015-04-17

**Authors:** Rachel Adihe Lokanga, Alireza Ghodsi Senejani, Joann Balazs Sweasy, Karen Usdin

**Affiliations:** 1 Section on Gene Structure and Disease, Laboratory of Cell and molecular Biology, National Institute of Diabetes, Digestive and Kidney Diseases, National Institutes of Health, Bethesda, Maryland, United States of America; 2 Department of Biochemistry, University of Cape Town Medical School, Cape Town, South Africa; 3 Departments of Therapeutic Radiology and Human Genetics, Yale University, School of Medicine, New Haven, Connecticut, United States of America; Duke University, UNITED STATES

## Abstract

The Fragile X-related disorders (FXDs) are members of the Repeat Expansion Diseases, a group of human genetic conditions resulting from expansion of a specific tandem repeat. The FXDs result from expansion of a CGG/CCG repeat tract in the 5’ UTR of the *FMR1* gene. While expansion in a FXD mouse model is known to require some mismatch repair (MMR) proteins, our previous work and work in mouse models of another Repeat Expansion Disease show that early events in the base excision repair (BER) pathway play a role in the expansion process. One model for repeat expansion proposes that a non-canonical MMR process makes use of the nicks generated early in BER to load the MMR machinery that then generates expansions. However, we show here that heterozygosity for a Y265C mutation in Polβ, a key polymerase in the BER pathway, is enough to significantly reduce both the number of expansions seen in paternal gametes and the extent of somatic expansion in some tissues of the FXD mouse. These data suggest that events in the BER pathway downstream of the generation of nicks are also important for repeat expansion. Somewhat surprisingly, while the number of expansions is smaller, the average size of the residual expansions is larger than that seen in WT animals. This may have interesting implications for the mechanism by which BER generates expansions.

## Author Summary

Unstable microsatellites are responsible for a number of debilitating human diseases known as the Repeat Expansion Diseases. The unstable microsatellites, which consist of tandem arrays of short repeat units, are prone to increase in length (expand) on intergenerational transmission and during the lifetime of the individual. Unlike the typical microsatellite instability seen in disorders like Lynch syndrome that arise from mutations in mismatch repair (MMR) genes, expansions of these microsatellites are abolished when MMR is lost. However, how MMR, which normally protects the genome against microsatellite instability, actually promotes microsatellite expansions in these diseases is unknown. There is evidence to suggest that a second DNA repair process, base excision repair (BER), may be involved, but whether the nicks generated early in the BER-process are subverted by an MMR-dependent pathway that generates expansions or whether some MMR proteins contribute to a BER-based expansion process is unclear. Here we show that a mutation that reduces the activity of Polβ, an essential BER enzyme, also reduces the expansion frequency. Since Polβ is essential for key events in BER downstream of the generation of nicks, our data favor a model in which expansions occur via a BER-dependent pathway in which MMR participates.

## Introduction

The Fragile X-related disorders (FXDs) are members of the group of diseases known as the Repeat Expansion Diseases. This group of diseases, which includes Huntington disease (HD) and Myotonic dystrophy type 1, are all caused by an increase in the number of repeats in an expansion-prone tandem repeat tract [1,2]. In the case of the FXDs the repeat is CGG/CCG and it is located in the 5’ untranslated region of the *FMR1* gene (MIM* 309550; reviewed in [3]). The FXDs include Fragile X-associated primary ovarian insufficiency and Fragile X-associated tremor/ataxia syndrome (MIM# 300623) that occur in carriers of alleles with 54–200 repeats, so-called premutation (PM) alleles. Fragile X syndrome (MIM# 300624), the leading heritable cause of intellectual disability is seen in carriers of full mutation alleles (>200 repeats).

The repeats responsible for the Repeat Expansion Diseases share the ability to form unusual secondary structures of one sort or another [1,2]. In the case of the FXDs, the repeats have the potential to form hairpins containing a mixture of Watson-Crick and Hoogsteen base pairs, as well as a variety of quadruplex structures [4,5,6,7,8,9,10]. Many of these sequences also form persistent RNA:DNA hybrids [11,12,13]. Current thinking in the field is that these structures are the substrates upon which the expansion and contraction processes act. However, the mechanism involved is unclear.

We have previously shown that oxidative damage exacerbates expansion risk in a mouse model of the FXDs [14]. Since Base Excision Repair (BER) is the major pathway involved in the repair of oxidized bases, this finding is consistent with the observation that OGG1 and NEIL1, DNA glycosylases involved in the initial recognition of oxidized bases in the BER pathway, are important for somatic expansion in a mouse model of HD [15,16]. However, the effect of DNA glycosylase mutations on intergenerational expansion was limited, with the loss of OGG1 having no effect, and the loss of NEIL1 reducing the average expansion size but not the expansion frequency. Whether this reflects mechanistic differences between germ line and somatic expansion or the contribution of other DNA glycosylases or other kinds of DNA damage to expansion is unclear. Furthermore, components of the mismatch repair (MMR) pathway have been shown to be essential for expansion in a number of different mouse and human tissue culture models of the Repeat Expansion Diseases [17,18,19,20,21,22,23,24,25,26]. This has led to the idea that BER *per se* does not lead to expansions but rather that the MMR machinery can use the nicks generated by BER DNA glycosylases to load MMR components onto the DNA that in turn are responsible for generating expansions via a non-canonical form of MMR [27].

To examine the contribution of downstream events in the BER pathway to repeat expansion we have examined the effect of a Y265C mutation in the gene encoding Polβ [28,29] on the expansion frequency in a mouse model of the FXDs. Polβ is the DNA polymerase central to BER. It acts downstream of the DNA glycosylases and the apurinic/apyrimidinic endonuclease 1 (APE1) to remove the 5′-terminal deoxyribose 5-phosphate resulting from APE1 digestion and in concert with DNA ligase 3 to complete the short patch pathway of BER. If short patch BER cannot be completed because of oxidation or reduction of the deoxyribose 5-phosphate then Polβ acts together with Lig1, FEN1 and sometimes other polymerases like Polδ and Polε, to repair the lesion using the long patch (LP) BER pathway. The Y265C mutation in *PolB* is a dominant mutation [30] that results in a Polβ with a normal lyase activity but a ~20-fold lower steady state catalytic rate than the WT enzyme and a lower fidelity [28].

We show here that this Polβ mutation causes a decrease in the frequency of expansions seen in the sperm of young males and decreases the extent of somatic instability in some tissues. Thus central events in the BER pathway that occur subsequent to the generation of a nick also play a role in repeat expansion in the FXD mouse. The distribution of residual expansions in these animals is biased towards larger expansions and this may reflect the use of alternative pathways for BER-mediated repeat expansion when Polβ activity is impaired.

## Results

### Heterozygosity for the *PolB* ^*c*^ allele causes a decrease in the number of expanded alleles detected in sperm

To examine the role of Polβ in repeat expansion we crossed our FX PM mice to mice with a Y265C mutation in *PolB* (*PolB* ^*c*^). Since we found homozygosity for the *PolB* ^*c*^ to be embryonic lethal in a pure C57BL/6 background and in animals backcrossed for 4 generations onto a 129S1 background, our efforts to understand the role of this protein in repeat expansion was of necessity limited to studying its effect in heterozygous animals. We confined our analysis to animals on a C57BL/6 background since there is reason to think that the expansion frequency would be higher in these animals [31]. Since mice heterozygous for a null mutation of *PolB* show significantly higher mutation rates in sperm than WT mice [32] and *PolB* ^*+/C*^ mice develop an autoimmune pathology that resembles systemic lupus erythematosus, a condition that has been suggested to reflect a BER deficiency [33], we rationalized that an effect of the Polβ mutation on expansion might be seen even in heterozygotes.

As the mutation rates in *PolB* ^+/-^ animals are only elevated significantly in sperm [32], we anticipated that the effect of heterozygosity for the *PolB* ^*C*^ mutation might be limited to sperm. We thus used small pool PCR analysis to investigate the effect of the *PolB* mutation on repeat expansion in the paternal gametes. In this process a pair of nested PCR reactions is carried out on sperm DNA that has been diluted such that on average one genome equivalent is present in the initial PCR reaction. Typically the second PCR reaction is positive for a PM allele <50% of the time and thus a product obtained in this way likely reflects the allele present in a single gamete. Multiple independent reactions allow the variants present in a population of alleles to be readily identified. This approach has been widely used for studying repeat instability in mice (reviewed in [34]) and humans with FX PM alleles [35].

**Fig 1:**
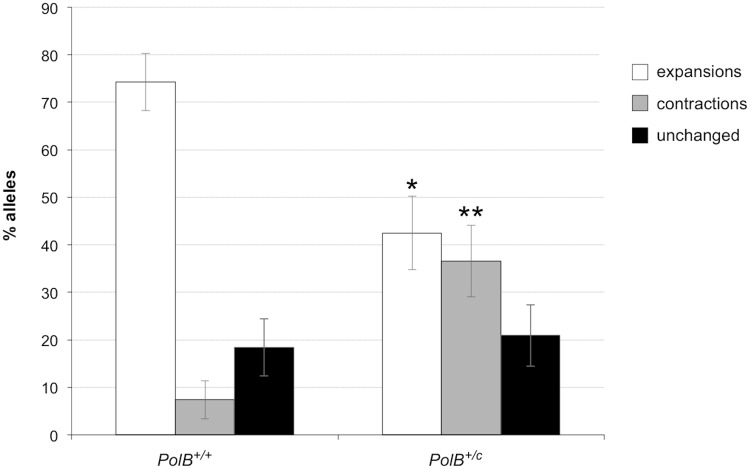
The effect of heterozygosity for the *PolBC* mutation on the number of expansions, contractions and unchanged alleles seen in the gametes of 3-month-old male mice. Small pool PCR was carried out on sperm DNA isolated from two 3-month-old *PolB+/+* and two 3-month-old *PolB+/C* male mice as described in the Materials and Methods. These animals all had ~140 repeats. The difference between the number of expansions, contractions and unchanged alleles within each genotype and between the two genotypes was evaluated by Fisher’s exact test. The error bars represent the 95% confidence intervals. There were no significant within genotype differences in the frequency of expansions, contractions or unchanged alleles. Allele classes that are significantly different in *PolB+/C* mice are marked with asterisks. Expansions were significantly reduced in *PolB+/C* gametes (p = 0.0001) and contractions significantly increased (p = 0.0001) by Fisher’s exact test.

**Fig 2:**
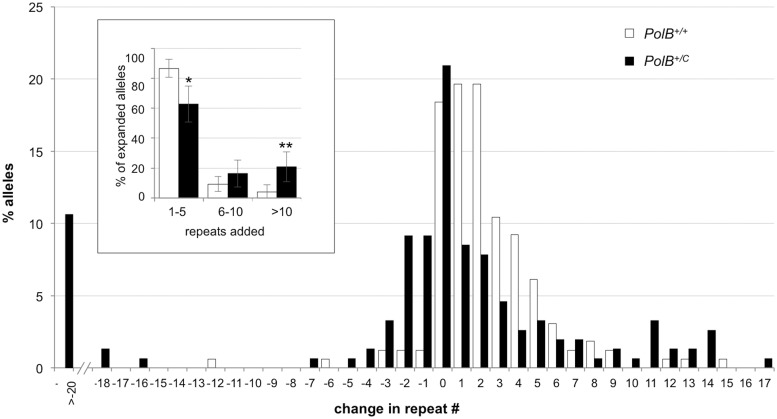
The effect of heterozygosity for the *PolBC* mutation on the distribution of repeat number changes seen in the gametes of 3-month-old male mice. The percentage of alleles with the indicated gains or losses in repeat number for *PolB+/+* and *PolB+/C* mice was plotted. The mean gain of repeats was 3.17 (SD = 2.52) for *PolB+/+* and 5.43 (SD = 4.48) for *PolB+/C*. This resulted in a distribution of expanded alleles that was significantly different in the two genotypes (p = 0.0001; *t* test). The mean loss of repeats was 10.83 (SD = 11.05) for *PolB+/+* and 19.68 (SD = 31.58) for *PolB+/C*. The very high standard deviations due to the presence of some very large contractions particularly in the *PolB+/C* mice resulted in a distribution of contracted alleles that was not significantly different in the two genotypes. Inset: *PolB+/C* mice have fewer small expansions and more large expansions than *PolB+/+* mice. The error bars represent the 95% confidence interval. Repeat size classes that are significantly different in *PolB+/C* mice are marked with an asterisk. The decrease in the number of alleles with 1–5 repeats was significant at p = 0.0001, and the increase in the number of alleles with >10 repeats was significant at p = 0.005.

As can be seen in Fig 1, the sperm of 3-month-old *PolB* ^*+/C*^ mice had a significantly lower number of expansions than the sperm of *PolB* ^*+/+*^ animals (Fig 1; 42% vs 74%; p = 0.0001). However, of the expansions that did occur, only 63% involved the addition of 1–5 repeats compared to 88% of the expansions seen in the sperm of WT mice (see Fig 2 inset). This relative deficit of small expansions is associated with a higher frequency of gametes that have gained >10 repeats: Only 3% of the expanded gametes of WT animals had gained this number of repeats while 22% of the expanded gametes of *PolB* ^*+/C*^ mice had done so. In addition, *PolB* ^*+/C*^ mice also had a 4.9-fold more gametes with alleles smaller than the parental allele (36.6% vs 7.4%, p = 0.0001). An increase in both large and small contractions was seen (Fig 2).

**Fig 3:**
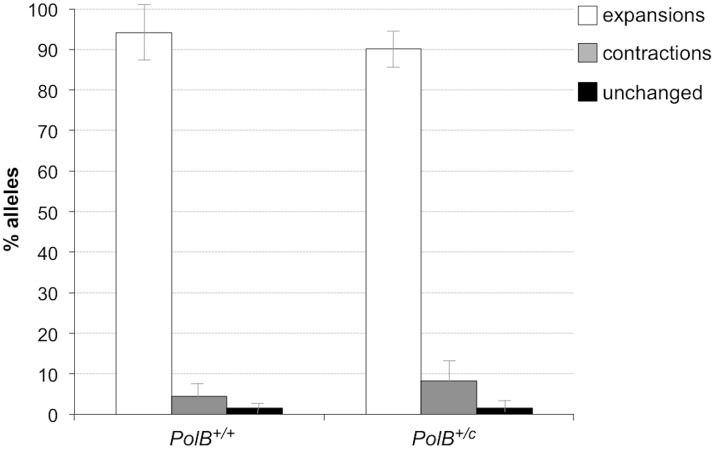
The effect of heterozygosity for the *PolBC* mutation on the number of expansions, contractions and unchanged alleles seen in the gametes of 11-month-old male mice. Small pool PCR was carried out on sperm DNA isolated from three 11-month-old *PolB+/+* and three 11-month-old *PolB+/C* male mice as described in the Materials and Methods. These animals all had ~140 repeats. The error bars represent the 95% confidence interval. The difference between the number of expansions, contractions and unchanged alleles in the two groups of animals was evaluated by Fisher’s exact test but no significant differences were found.

**Fig 4:**
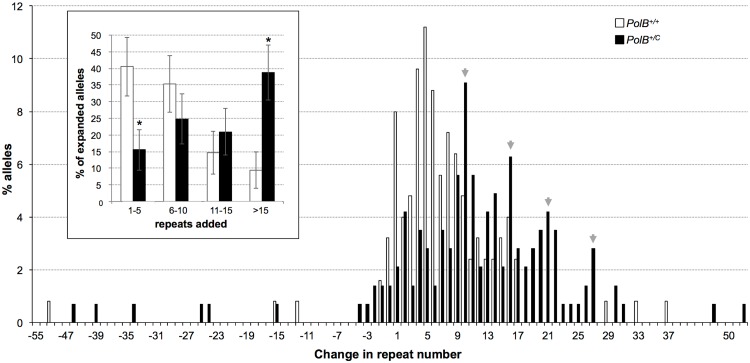
The effect of heterozygosity for the *PolBC* mutation on the distribution of repeat number changes seen in the gametes of 11-month-old male mice. The percentage of alleles with the indicated change in repeat number that were seen in the gametes of three 11-month-old mice *PolB+/+* and three 11-month old *PolB+/C* mice. The grey arrowheads indicate the local maxima seen in the distribution of *PolB+/C* alleles. The mean gain of repeats was 8.75 (SD = 5.96) for *PolB+/+* and 14.04 (SD = 8.70) for *PolB+/C*. This resulted in a distribution of expanded alleles that was significantly different in the two genotypes (p = 0.0001; *t* test). Too few contractions were seen to carry out any statistical analysis. Inset: *PolB+/C* mice have fewer small expansions and more large expansions than *PolB+/+* mice. The error bars represent the 95% confidence interval. Repeat size classes that are significantly different in *PolB+/C* mice are marked with an asterisk. The decrease in the number of alleles with 1–5 repeats was significant at p = 0.0001, and the increase in the number of alleles with >15 repeats was also significant at p = 0.0001.

When small pool PCR was carried out on the sperm of 11-month-old animals, no significant difference in the number of expansions was seen in the gametes of *PolB* ^+/+^ and *PolB* ^*+/C*^ mice (Fig 3). This was not unexpected since we had previously shown that the number of expansions in the germ line increases with paternal age [36]. Since the number of gametes that had sustained at least one expansion in *PolB* ^*+/C*^ mice was 42% at 3 months of age, it was not surprising that this number had risen to ~90% by 11 months of age. However, when we examined the distribution of residual expansions in these animals, we were surprised to see a relative enrichment for larger alleles (Fig 4). In WT animals the average number of repeats added had increased from an average of ~1 repeat in 3 month old mice to an average of ~7 repeats in the older animals consistent with our previous reports [36]. In contrast, in *PolB* ^*+/C*^ mice, a similar deficit of smaller alleles was seen in older mice as was seen in younger ones. In addition, the number of larger alleles had increased such that 39% of gametes that had expanded had gained more than 15 repeats compared to 9% in *PolB* ^*+/+*^ animals. This data would be consistent with the idea that while the expansion frequency is lower in *PolB* ^*+/C*^ mice than it is in *PolB* ^*+/+*^ animals, when expansions do occur, they tend to be larger. Thus repeated rounds of expansion in the germ line of *PolB* ^*+/+*^ males would, for the most part, lead to an incremental increase in repeat number with time, while in *PolB* ^*+/C*^ mice, each expansion event, while less frequent, would add a larger number of repeats. The distribution of allele sizes in the gametes of 11-month-old *PolB* ^*+/C*^ mice showed a series of local maxima corresponding to gametes with 10, 16, 21 and 27 added repeats (indicated by the gray arrowheads in Fig 4). Taken together with the data for 3 month old fathers, the pattern would be consistent with many gametes in *PolB* ^*+/C*^ mice having undergone multiple rounds of expansion each involving the addition of ~5–6 repeats, whereas in *PolB* ^*+/C*^ mice most expansions only add ~1 repeat.

The number of alleles that were smaller than the original paternal allele was 8.4% in the gametes of 11-month-old *PolB* ^*+/C*^ mice (Fig 3). This represents a significant decrease relative to the number of such alleles seen in younger animals. In contrast the number of smaller alleles in *PolB* ^*+/+*^ mice did not change significantly with age. The reduction in the number of smaller alleles in mutant mice would be consistent with the idea that between the ages of 3 and 11 months most gametes in *PolB* ^*+/C*^ mice that had initially undergone a contraction subsequently underwent one or more rounds of expansion.

### Heterozygosity for the *PolB* ^*c*^ allele also causes a decrease in the extent of somatic expansion in brain and tail

To evaluate the effect of the Y265C mutation on somatic expansion we compared the extent of expansion in different tissues of 16-month-old *PolB* ^*+/+*^ and *PolB* ^*+/C*^ male mice. The extent of expansion in individual tissues from each group of mice was quantified using the previously described Somatic Instability Index (SII) [37]. This index is based on the sum of the relative heights of the individual peaks seen in high resolution electropherograms generated from the products of amplification across the repeats. It thus can be used to quantitate the extent of expansion in a given tissue with age or relative to the same tissue in the neonate or an organ like heart that shows very little expansion.

**Fig 5:**
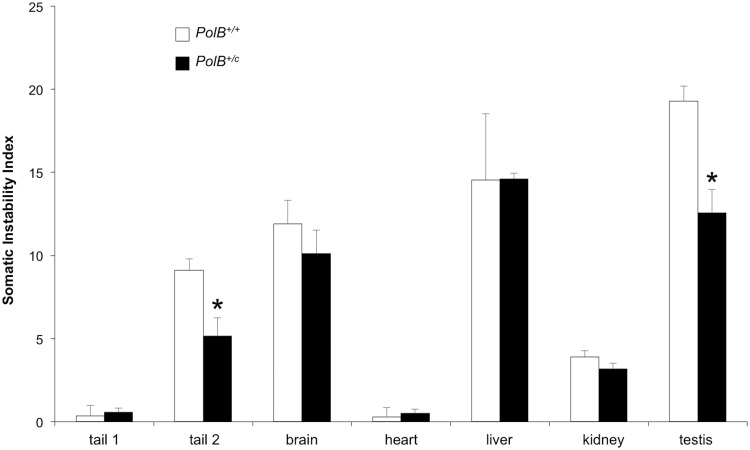
PolB+/C mice show a reduced somatic instability index in testis and tail. The somatic instability index of different organs of three 16 month old *PolB+/+* and three 16 month old *PolB+/C* mice with ~140 repeats was determined as previously described [46]. Tail 1 and tail 2 refer to tail samples taken at 3 weeks of age and tail samples taken at 16 months respectively. The error bars represent the standard deviations. The significance of the differences in the SII for different genotypes was determined using Student’s t-test. The tissues in which the SII was significantly lower in *PolB+/C* mice are indicated by asterisks. The SII for *PolB+/C* testis was significantly lower at p = 0.001 and the SII for the *PolB+/C* tail 2 sample was significantly lower at p = 0.013.

As can be seen from Fig 5, some tissues of *PolB* ^*+/C*^ mice have an average SII that is lower than their WT counterparts. The difference in SII was significant for testis, and the tail sample taken at euthanasia (Tail 2). While the expansion frequency seen in sperm of 11-month-old animals is not significantly different from WT animals, the lower SII seen in the testis as a whole may reflect the contribution of other cells of the testis. The limited number of tissues affected by the presence of the *PolB* ^*C*^ mutation is consistent with the limited effect of *PolB* mutations on the mutation rates of different tissue [32], an observation that has been interpreted to mean that DNA repair in most tissues is not sensitive to *PolB* ^*C*^ heterozygosity. Since we know that contractions do not occur post-natally in somatic cells [38], the decrease in the SII would be consistent with a role for Polβ in generating somatic expansions. While it is possible that some expansions seen in testis are derived from developing gametes, the fact that a decreased SII is also seen in tail indicates that the effect of the Polβ mutation is not confined to germ cells. While in sperm a lower expansion frequency is offset by a larger average expansion size, this effect is not apparent in either total testis DNA or tail. It may be that the decrease in the expansion frequency caused by the Y265C mutation is more marked in these cells than it is in sperm, such that the larger jumps produced by those few alleles that do expand is not apparent. Alternatively, the pathway that generates these larger jumps may not occur at high enough frequency for the effect to be seen in somatic cells.

### Relationship between proteins involved in BER and the propensity of some organs to show high levels of expansion

**Fig 6:**
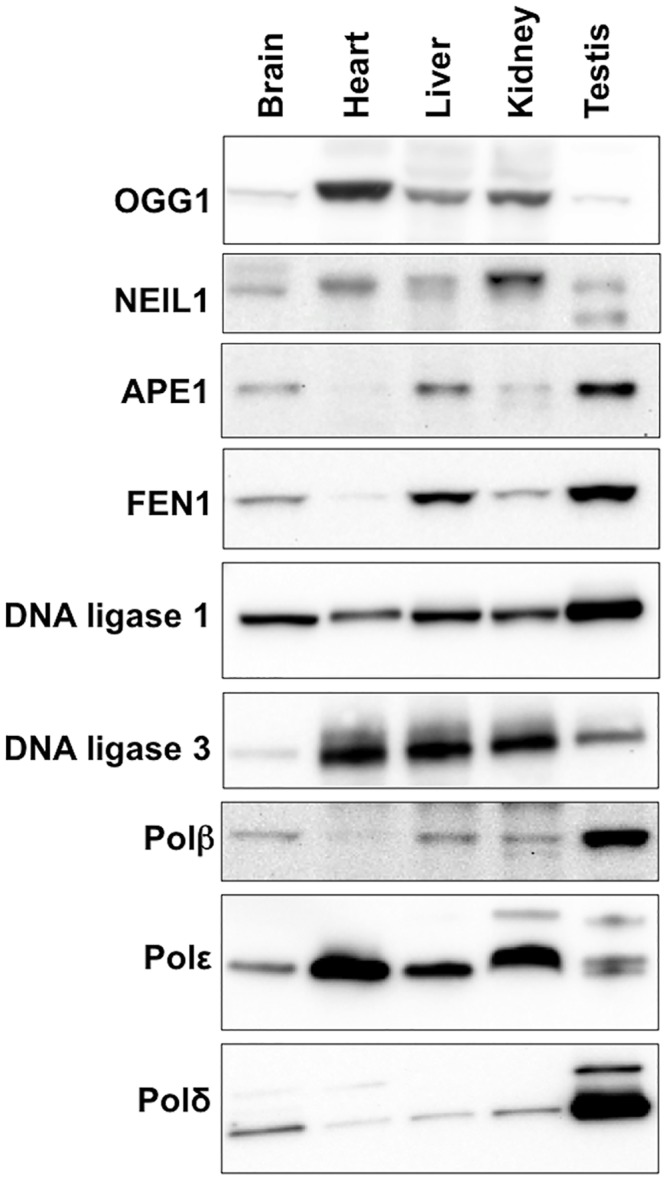
Expression of various BER proteins in different mouse organs. Total protein was extracted from different organs of 3 different FXD mice as described in the Materials and Methods. Since in our experience proteins used as “normalizing controls” including β-actin and α-tubulin and GAPDH differ significantly in different organs, we took care to analyze equal amounts of protein as assessed by the Bradford Assay. Ten micrograms of protein from the organs of each animal were pooled and loaded onto 3–8% Tris-Acetate gels, resolved by gel electrophoresis and subjected to Western blotting as described in the Materials and Methods.

The propensity of the CAG/CTG repeat to expand more in striatum than in the cerebellum of a mouse model for HD has been attributed to differences in the stoichiometry of the proteins involved in BER that are expressed in these two brain regions [39,40]. To evaluate the role of these proteins in determining the tissue specificity of expansion in the Fragile PM mouse we compared the expression levels of the key BER enzymes APE1, DNA ligase 1, DNA ligase 3, FEN1, OGG1, NEIL1 and Polβ in a selection of different tissues. Expansion levels in these tissues follow the trend: testis (SII = 19) > liver (SII = 15) > brain (SII = 12) > kidney (SII = 4) > heart (SII = 0). As can be seen from Fig 6, some of the proteins tested do not show a good correlation with the propensity to expand. For example, DNA ligase 3 and OGG1 show the highest levels of expression in heart, an organ in which the repeat is stable whilst NEIL1 is expressed at its lowest levels in brain, liver and testis, organs that show the highest levels of expansion. Furthermore, organs that have very different propensities to expand, like brain and heart, have similar levels of Polβ. A low level of FEN1 relative to Polβ has been suggested to be an important determinant of the predisposition of cells of the striatum to expand relative to cells of the cerebellum in a HD mouse model [41]. However, in our mouse background this relationship does not appear to hold, with the FEN1/Polβ ratio being lower in heart, an organ that shows no expansion, than it is in brain. Of all the proteins tested, only APE1 and FEN1 showed a general correlation between the level of expression and the SII of all 5 organs tested.

## Discussion

Here we demonstrate that heterozygosity for the Polβ Y265C mutation causes a significant decrease in the number of expansions seen in the paternal germ line of young FX PM mice (Fig 1). This decrease specifically impacted the class of repeat length changes involving the addition of 1–5 repeats, while not negatively impacting larger repeat expansions (Fig 2). In fact, in both 3-month-old and 11-month-old *PolB* ^*+/C*^ males the number of gametes with larger expansions was significantly higher than it was in WT animals (Figs 2 and 4). The Y265C mutation also resulted in a decrease in somatic expansion that was significant in DNA from testis and tail but not other organs tested like liver and brain (Fig 5). The differential effect of heterozygosity for the *PolB* ^*c*^ mutation on the extent of somatic expansion in different tissue does not necessarily mean that different mechanisms of expansion operate in different organs. Evidence from mice heterozygous for a null allele of *PolB* demonstrate that liver and brain do not show reduced levels of Polβ or increased levels of mutation, while male germ cells do [32]. This may be because some cells have regulatory mechanisms that are able to compensate for the presence of only one fully functional allele.

While 3 month old *PolB* ^*+/C*^ mice showed evidence of more contractions than *PolB* ^*+/+*^ mice (Figs 1 and 2), at 11 months of age the number of alleles that were smaller than the original parental allele in the *PolB* ^*+/C*^ mice were the same as those seen in age-matched *PolB* ^*+/+*^ mice (Figs 3 and 4). This would be consistent with the hypothesis that when BER is suboptimal, a higher than normal fraction of alleles are processed by an alternative, currently unidentified, repair pathway that leads to contractions, but that over time the number of alleles smaller than the parental allele decreases as those alleles undergo subsequent rounds of expansion. These data also suggest that the *PolB* ^*C*^ mutation does not reduce the frequency of intergenerational expansions by promoting contractions, but rather that it directly impacts the efficacy of the expansion process. Thus these data implicate Polβ, and thus central events in the BER pathway, in generating expansions.

**Fig 7:**
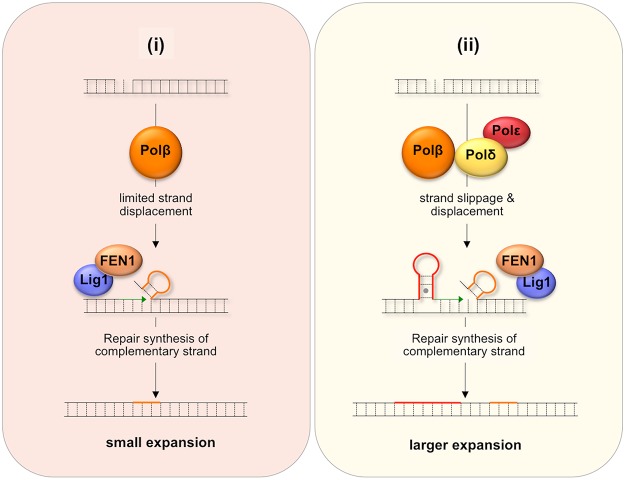
Model for BER-mediated repeat expansion in the FX PM mouse. Nicks that do not get repaired by short patch BER may be channeled into one of two branches of the LP BER pathway. (*i) The Polβ-dependent*, *Polδ/Polε-independent branch*. Nick processing is carried out by Polβ, a poorly processive polymerase with weak strand-displacement activity. The resultant small flaps are processed by FEN1 to generate a ligatable 5’ end that still contains a few additional flap bases (shown in orange). (ii) The *Polβ/Polδ/Polε-dependent branch*. Since both Polδ and Polε are more processive than Polβ, more strand slippage and more extensive strand displacement may result. Repriming of DNA synthesis on the slipped-strand using Polβ would not remove looped out bases (shown in red) since Polβ lacks a suitable proofreading activity. Limited strand displacement by Polβ or more extensive strand displacement by Polδ/Polε, followed by FEN1 cleavage could also result a ligatable end that still contains some flap bases (shown in orange). In either case, repair synthesis initiated on the complementary strand would fix the supernumerary bases into the derivative allele thus generating either a small (i) or large (ii) expansion. MMR proteins may facilitate this process by stabilizing the hairpins. These proteins may also directly generate expansions by channelling the hairpins formed during BER into the MMR pathway.

One proposed model for BER-mediated repeat expansion suggests that expansion results from a Polβ/FEN1-dependent branch of the LP BER pathway where the weak strand displacement synthesis activity of Polβ is proposed to facilitate limited strand-slippage of the repeats in the DNA downstream of the nick [42] as illustrated in Fig 7(i). This process would be promoted by hairpin formation by the repeats and would create a larger gap that would need to be filled by Polβ. A stable hairpin would force FEN1 to capture and cleave a series of short flaps resulting from breathing or realignment of the 5’ end of the hairpins. This so-called alternate cleavage process would be essential for the creation of the ligatable nick necessary to complete repair. The failure to fully remove the flap would result in expansions. A second model, illustrated in Fig 7(ii), proposes that expansion arises via the use of a second branch of the LP BER pathway in which DNA synthesis is carried out by a combination of Polβ, Polδ and perhaps Polε. Expansion would be triggered by strand slippage of the nascent strand during progressive DNA synthesis by Polδ/Polε and the resultant formation of a hairpin by the repeats [43]. If the hairpin does not have a 3’ tail, Polδ and Polε could reinitiate DNA synthesis after using their 3’-5’ proofreading activity to remove the hairpin. However, if Polβ reinitiates synthesis the hairpin would be retained since Polβ has no such proofreading activity. This would result in repeat expansion if the hairpin were not subsequently removed. Some strand displacement could also contribute to the incorporation of additional bases.

It may be that the differential effect of the Y265C mutation on the frequency of small and large expansions can be ascribed to the contribution of both branches of the LP BER pathway to repeat expansion if one were to generate small expansions and the other larger ones. For example, it may be that a Polδ/Polε-independent branch that is dependent on the weak strand-displacement activity of Polβ to generate supernumerary bases would give rise to small expansions as illustrated in Fig 7(i), while the Polβ/Polδ/Polε branch could give rise to larger expansions as shown in Fig 7(ii), since Polδ/Polε could generate hairpins both by strand slippage and strand displacement [44]. In this view, the slower polymerization rate of the Y265C mutant would contribute to the reduction in the overall expansion frequency and to the frequency of small expansions via the Polδ/Polε-independent branch. The slower polymerization rate may reduce the extent of strand displacement by Polβ and give FEN1 longer to properly process the flap bases [42]. The Y265C mutation may be less likely to negatively impact the use of the pathway in which Polδ/Polε also participates and thus may not reduce the fraction of alleles that sustain larger expansions.

In many mouse models of the Repeat Expansion Diseases smaller increases in repeat number are characteristic of tissue such as kidney or tail while larger increases in repeat number are more typical of liver and some parts of the brain (see [38,45,46] for examples). It is tempting to speculate that these differences result from the differential use of these two branches of the LP BER pathway with the pathway choice perhaps being related to the relative levels of Polβ and Polδ/Polε.

LP BER requires Ligase 1 and FEN1. In previous work we showed that there was no effect of either *Fen1* heterozygosity or homozygosity for a *Lig1* hypomorphic mutation on the frequency of repeat expansion in our mouse model [14]. However, since *Fen1* and *Lig1* null mice are not viable, a role for these proteins in repeat expansion could not be definitely excluded. The requirement of MSH2 for repeat expansion in this mouse model [19] may reflect a role of MSH2-containing complexes in binding to and stabilizing structures formed by strand slippage or strand displacement thus favoring the incorporation of supernumerary bases into the “repaired” strand. It is also possible that this binding leads to the recruitment of the rest of the MMR machinery to carry out BER-dependent, MMR-generated expansions.

There was not a simple relationship between the level of expression of proteins active in BER like OGG1, NEIL1, Lig1, and Polβ in different mouse organs and the extent of somatic expansion. Nor were the highest levels of expansion associated with organs that showed the lowest FEN1/Polβ ratio as previously suggested [47]. However, a correlation was seen between the level of expression of both APE1 and FEN1 and the amount of expansion seen in a particular tissue. Specifically, the levels of these proteins increased in the order heart<kidney<brain<liver<testis, a progression that parallels the increase in the SII of those organs (Figs 5 and 6). In particular, the correlation with APE1 expression may be of significance since APE1 facilitates loading of Polβ on the incised AP site and stimulates the strand-displacement activity of Polβ, thus facilitating the use of the LP BER pathway [41]. The correlation between APE1 expression and the propensity to expand is interesting since the abasic sites that are substrates for APE1 can be generated independently of DNA glycosylase activity. For example, it is thought that spontaneous depurination, to which the G-rich FX repeats may be particularly prone [48], is one of the most frequent promutagenic events that impacts the mammalian genome [49]. The association between elevated APE1 levels and the likelihood of expansion may thus point to sources of DNA damage that in addition to oxidative stress, may contribute to expansion risk.

We have also previously shown that expansion only occurs when the PM allele is located on the active X chromosome in the FX PM mouse [50]. A similar dependence for the PM allele to be on the active X is also suggested by data from humans [51]. However, we have also shown that while CSB, a protein essential to the Transcription Coupled Repair pathway, contributes to expansions it likely does so via a different mechanism [36]. Since it has been reported that BER complexes in general [52] and LP BER complexes in particular [53] are excluded from heterochromatic regions of the genome, the use of the LP BER pathway may account for this dependence.

## Materials and Methods

### Mouse breeding and maintenance

The FX PM and the *PolB* ^c^ mice were generated as previously described [29,54]. Mice were maintained in accordance with the guidelines of the NIDDK Animal Care and Use Committee and with the *Guide for the Care and Use of Laboratory Animals* (NIH publication no. 85–23, revised 1996). No *PolB* ^*c/c*^ mice were ever obtained from *PolB* ^*+/C*^ crosses in the C57BL/6 background or after backcrossing for more than four generations onto a 129S1/SvImJ background. Since there is data to suggest that there would be fewer expansions in 129S1 background than a C57BL/6 background [31], we therefore confined our analysis to heterozygous animals in the C57BL/6 background.

### DNA isolation and genotyping

Genomic DNA from tails and different mouse organs was extracted using a Maxwell®16 Mouse tail DNA purification kit (Promega, Madison, WI) according to the manufacturer’s instructions. Sperm was isolated from mouse epididymis using standard procedures. Polβ genotyping was carried out by PCR analysis of tail DNA using the forward primer Y265C-F1: 5′AGAAAAGCAGCTTCCAGCAG and reverse primer Y265C-R4: 5′CAGACTTTCCAAGTGCAGGAT. PCR was carried out at 95°C for 3 min followed by 30–40 cycles of 95°C denaturation for 30 s, 60°C annealing for 15 s, and 72°C extension for 1 min. The WT allele produced a 440 bp PCR product and the mutant a 490 bp product [29]. The presence of the expanded CGG•CCG-repeat tract was determined as described previously using the frax-m4 (5’-CTTGAGGCCCAGCCGCCGTCGGCC-3’) and frax-m5 (5’-CGGGGGGCGTGCGGTAACGGCCCAA-3’) primers [55]. The primer binding sites are located immediately adjacent to the repeat and the PCR product corresponds to the length of the repeat with 49 bases of flanking sequence. For genotyping the PCR products were resolved by electrophoresis on a 1.5% agarose gel and stained with ethidium bromide.

### Repeat analysis

For determination of the repeat number the repeat was amplified using as described previously except that primer frax-m4 was labeled with 6-carboxyfluorescein (FAM) [55]. For determination of the repeat number or profile in bulk DNA, 50–100 ng of DNA was used for the PCR. For small pool PCR analysis from sperm, the DNA was diluted to 3pg/μl (roughly 1 haploid genome equivalent/μl). The diluted DNA was then subjected to nested PCR. The first round of PCR was carried out using the primers frax-C (5’-gctcagctccgtttcggtttcacttccggt-3’) and frax-F (5’-agccccgcacttccaccaccagctcctcca-3’) in a 25 μl reaction using the same PCR conditions used previously [55]. One microliter of this PCR reaction was then subjected to a second round of PCR using frax-m4 and frax-m5. Our PCR conditions allow us to amplify a wide range of repeat lengths without significant allele dropout or bias. Under these conditions <50% of samples yielded a PCR product. This is consistent with the idea that each positive PCR reaction likely represented the products of amplification of DNA from a single sperm cell. The total number of positive PCR reactions was considered to represent the total number of alleles analyzed. The PCR products were resolved by capillary electrophoresis on an ABI Genetic Analyzer and the PCR profiles analyzed using GeneMapper Software 5. Alleles that were smaller, larger or the same size as the parental allele were counted and the proportion of each allele size class calculated as a fraction of the total number of alleles analyzed. The somatic instability index from different organs was calculated using 3 mice of each genotype as previously described [36]. Statistical analysis of these data were carried out using the Graphpad Quickcalcs website (http://www.graphpad.com/quickcalcs/). The 95% confidence intervals for the proportion of expansions, contractions and unchanged alleles were determined using the Graphpad implementation of the modified Wald method. The significance of the differences in the frequency with which these classes were observed in *PolB* ^*+/+*^ mice and *PolB* ^*+/C*^ mice was determined using Fisher’s exact test. The significance of the differences in the SII and the distribution of derivative alleles in gametes was determined using Student’s t-test.

### Western blotting

Protein extracts were prepared from flash frozen tissues. Tissues were homogenized using a tissue homogenizer (Precellys® 24,Bertin Technologies, Rockville, MD) with T-PER protein extraction reagent (Pierce Biotechnology, Inc, Rockford, IL) supplemented with complete, Mini, EDTA-free protease inhibitor cocktail (Roche Applied Science, Indianapolis, IN) and phosphatase inhibitor cocktail-3 (Sigma-Aldrich, St. Louis, MO) according to the supplier’s instructions. The protein concentration was determined using a Bio-Rad protein assay kit (Bio-Rad, Hercules, CA). Because of tissue-specific differences in the expression of proteins often used as normalizing controls, including β-actin, tubulin and GAPDH, care was taken to load equal amounts of protein in each gel lane. Prior to loading, the protein samples were heated for 10 min at 70°C in LDS-Sample Buffer (Life Technologies, Grand Island, NY) supplemented with 50 mM DTT. The proteins were then resolved by electrophoresis on 3–8% NuPAGE Novex Tris-Acetate gels (Life Technologies) and electro-blotted onto nitrocellulose membranes using NuPAGE Transfer Buffer (Life Technologies) supplemented with 10% methanol. Transfer was carried out at 100 V at room temperature for 1 hour. Membranes were blocked for one hour at room temperature in 5% ECL Prime blocking agent (GE Healthcare Bio-Sciences, Pittsburgh, PA) in TBST pH 7.5 (10 mM Tris-HCl, 0.15 mM NaCl and 0.01% Tween 20), then incubated overnight at 4°C with antibodies to the following proteins APE1 (ab137708), DNA ligase 1 (ab76232), FEN1 (ab70815); NEIL1 (ab21337) and Polβ (ab26343) from Abcam (Cambridge, MA); OGG1 (15125-1-AP) from Proteintech Group (Chicago, IL) and DNA ligase 3 (611876) from BD Biosciences (Sparks, MD). After incubation with the appropriate secondary antibody and the addition of the ECL Prime detection reagent (GE Healthcare Bio-Sciences), the blot was imaged using a Fluorchem M imaging system (Proteinsimple, Santa Clara, CA).

## References

[1] Mirkin SM (2006) DNA structures, repeat expansions and human hereditary disorders. Current Opinion in Structural Biology 16: 351–358. 16713248 10.1016/j.sbi.2006.05.004

[2] Fry M, Usdin K (2006) Human Nucleotide Expansion Disorders; Gross H, editor. Heidelberg: Springer.

[3] Chonchaiya W, Schneider A, Hagerman RJ (2009) Fragile X: a family of disorders. Adv Peds 56: 165–186. 10.1016/j.yapd.2009.08.008 PMC2921504 19968948

[4] Fry M, Loeb LA (1994) The fragile X syndrome d(CGG)n nucleotide repeats form a stable tetrahelical structure. Proc Natl Acad Sci U S A 91: 4950–4954. 8197163 10.1073/pnas.91.11.4950 PMC43907

[5] Renciuk D, Zemanek M, Kejnovska I, Vorlickova M (2009) Quadruplex-forming properties of FRAXA (CGG) repeats interrupted by (AGG) triplets. Biochimie 91: 416–422. 10.1016/j.biochi.2008.10.012 19028545

[6] Usdin K (1998) NGG-triplet repeats form similar intrastrand structures: implications for the triplet expansion diseases. Nucleic Acids Res 26: 4078–4085. 9705522 10.1093/nar/26.17.4078 PMC147794

[7] Usdin K, Woodford KJ (1995) CGG repeats associated with DNA instability and chromosome fragility form structures that block DNA synthesis in vitro. Nucleic Acids Res 23: 4202–4209. 7479085 10.1093/nar/23.20.4202 PMC307363

[8] Mitas M, Yu A, Dill J, Haworth IS (1995) The trinucleotide repeat sequence d(CGG)15 forms a heat-stable hairpin containing Gsyn. Ganti base pairs. Biochemistry 34: 12803–12811. 7548035 10.1021/bi00039a041

[9] Yu A, Barron MD, Romero RM, Christy M, Gold B, et al (1997) At physiological pH, d(CCG)15 forms a hairpin containing protonated cytosines and a distorted helix. Biochemistry 36: 3687–3699. 9132022 10.1021/bi9625410

[10] Fojtik P, Vorlickova M (2001) The fragile X chromosome (GCC) repeat folds into a DNA tetraplex at neutral pH. Nucleic Acids Res 29: 4684–4690. 11713318 10.1093/nar/29.22.4684 PMC92515

[11] Loomis EW, Sanz LA, Chedin F, Hagerman PJ (2014) Transcription-Associated R-Loop Formation across the Human FMR1 CGG-Repeat Region. PLoS Genet 10: e1004294 10.1371/journal.pgen.1004294 24743386 PMC3990486

[12] Groh M, Lufino MM, Wade-Martins R, Gromak N (2014) R-loops Associated with Triplet Repeat Expansions Promote Gene Silencing in Friedreich Ataxia and Fragile X Syndrome. PLoS Genet 10: e1004318 10.1371/journal.pgen.1004318 24787137 PMC4006715

[13] Grabczyk E, Mancuso M, Sammarco MC (2007) A persistent RNA.DNA hybrid formed by transcription of the Friedreich ataxia triplet repeat in live bacteria, and by T7 RNAP in vitro. Nucleic Acids Res 35: 5351–5359. 17693431 10.1093/nar/gkm589 PMC2018641

[14] Entezam A, Lokanga AR, Le W, Hoffman G, Usdin K (2010) Potassium bromate, a potent DNA oxidizing agent, exacerbates germline repeat expansion in a fragile X premutation mouse model. Hum Mutat 31: 611–616. 10.1002/humu.21237 20213777 PMC2951473

[15] Kovtun IV, Liu Y, Bjoras M, Klungland A, Wilson SH, et al (2007) OGG1 initiates age-dependent CAG trinucleotide expansion in somatic cells. Nature 447: 447–452. 17450122 10.1038/nature05778 PMC2681094

[16] Mollersen L, Rowe AD, Illuzzi JL, Hildrestrand GA, Gerhold KJ, et al (2012) Neil1 is a genetic modifier of somatic and germline CAG trinucleotide repeat instability in R6/1 mice. Human molecular genetics 21: 4939–4947. 10.1093/hmg/dds337 22914735 PMC3607484

[17] Foiry L, Dong L, Savouret C, Hubert L, te Riele H, et al (2006) Msh3 is a limiting factor in the formation of intergenerational CTG expansions in DM1 transgenic mice. Hum Genet 119: 520–526. 16552576 10.1007/s00439-006-0164-7

[18] Kovtun IV, McMurray CT (2001) Trinucleotide expansion in haploid germ cells by gap repair. Nat Genet 27: 407–411. 11279522 10.1038/86906

[19] Lokanga RA, Zhao X-N, Usdin K (2014) The mismatch repair protein, MSH2, is rate-limiting for repeat expansion in a Fragile X premutation mouse model. Hum Mutat 35: 129–136. 24130133 10.1002/humu.22464 PMC3951054

[20] Manley K, Shirley TL, Flaherty L, Messer A (1999) Msh2 deficiency prevents in vivo somatic instability of the CAG repeat in Huntington disease transgenic mice. Nat Genet 23: 471–473. 10581038 10.1038/70598

[21] Savouret C, Brisson E, Essers J, Kanaar R, Pastink A, et al (2003) CTG repeat instability and size variation timing in DNA repair-deficient mice. EMBO J 22: 2264–2273. 12727892 10.1093/emboj/cdg202 PMC156074

[22] Gomes-Pereira M, Fortune MT, Ingram L, McAbney JP, Monckton DG (2004) Pms2 is a genetic enhancer of trinucleotide CAG.CTG repeat somatic mosaicism: implications for the mechanism of triplet repeat expansion. Hum Mol Genet 13: 1815–1825. 15198993 10.1093/hmg/ddh186

[23] Du J, Campau E, Soragni E, Ku S, Puckett JW, et al (2012) Role of mismatch repair enzymes in GAA.TTC triplet-repeat expansion in Friedreich ataxia induced pluripotent stem cells. J Biol Chem 287: 29861–29872. 10.1074/jbc.M112.391961 22798143 PMC3436184

[24] Halabi A, Ditch S, Wang J, Grabczyk E (2012) DNA mismatch repair complex MutSbeta promotes GAA.TTC repeat expansion in human cells. J Biol Chem 287: 29958–29967. 10.1074/jbc.M112.356758 22787155 PMC3436174

[25] Gannon AM, Frizzell A, Healy E, Lahue RS (2012) MutSbeta and histone deacetylase complexes promote expansions of trinucleotide repeats in human cells. Nucleic Acids Res 40: 10324–10333. 10.1093/nar/gks810 22941650 PMC3488247

[26] Pinto RM, Dragileva E, Kirby A, Lloret A, Lopez E, et al (2013) Mismatch repair genes Mlh1 and Mlh3 modify CAG instability in Huntington's disease mice: genome-wide and candidate approaches. PLoS Genet 9: e1003930 10.1371/journal.pgen.1003930 24204323 PMC3814320

[27] Pena-Diaz J, Bregenhorn S, Ghodgaonkar M, Follonier C, Artola-Boran M, et al (2012) Noncanonical mismatch repair as a source of genomic instability in human cells. Mol Cell 47: 669–680. 10.1016/j.molcel.2012.07.006 22864113

[28] Washington SL, Yoon MS, Chagovetz AM, Li SX, Clairmont CA, et al (1997) A genetic system to identify DNA polymerase beta mutator mutants. Proc Natl Acad Sci U S A 94: 1321–1326. 9037051 10.1073/pnas.94.4.1321 PMC19789

[29] Senejani AG, Dalal S, Liu Y, Nottoli TP, McGrath JM, et al (2012) Y265C DNA polymerase beta knockin mice survive past birth and accumulate base excision repair intermediate substrates. Proc Natl Acad Sci U S A 109: 6632–6637. 10.1073/pnas.1200800109 22493258 PMC3340078

[30] Clairmont CA, Sweasy JB (1998) The Pol beta-14 dominant negative rat DNA polymerase beta mutator mutant commits errors during the gap-filling step of base excision repair in Saccharomyces cerevisiae. J Bact 180: 2292–2297. 9573177 10.1128/jb.180.9.2292-2297.1998 PMC107167

[31] Tome S, Manley K, Simard JP, Clark GW, Slean MM, et al (2013) MSH3 polymorphisms and protein levels affect CAG repeat instability in Huntington's disease mice. PLoS genetics 9: e1003280 10.1371/journal.pgen.1003280 23468640 PMC3585117

[32] Allen D, Herbert DC, McMahan CA, Rotrekl V, Sobol RW, et al (2008) Mutagenesis is elevated in male germ cells obtained from DNA polymerase-beta heterozygous mice. Biol Reprod 79: 824–831. 10.1095/biolreprod.108.069104 18650495 PMC2679517

[33] Ray S, Menezes MR, Senejani A, Sweasy JB (2013) Cellular roles of DNA polymerase beta. Yale J Biol Med 86: 463–469. 24348210 PMC3848100

[34] Gomes-Pereira M, Bidichandani SI, Monckton DG (2004) Analysis of unstable triplet repeats using small-pool polymerase chain reaction. Methods Mol Biol 277: 61–76. 15201449 10.1385/1-59259-804-8:061

[35] Crawford DC, Wilson B, Sherman SL (2000) Factors involved in the initial mutation of the fragile X CGG repeat as determined by sperm small pool PCR. Hum Mol Genet 9: 2909–2918. 11092767 10.1093/hmg/9.19.2909

[36] Zhao X-N, Usdin K (2014) Gender and cell-type specific effects of the transcription coupled repair protein, ERCC6/CSB, on repeat expansion in a mouse model of the Fragile X-related disorders. Hum Mutat 35: 341–349. 24352881 10.1002/humu.22495 PMC4067466

[37] Lee JM, Zhang J, Su AI, Walker JR, Wiltshire T, et al (2010) A novel approach to investigate tissue-specific trinucleotide repeat instability. BMC Syst Biol 4: 29 10.1186/1752-0509-4-29 20302627 PMC2856555

[38] Lokanga RA, Entezam A, Kumari D, Yudkin D, Qin M, et al (2013) Somatic expansion in mouse and human carriers of Fragile X premutation alleles. Hum Mutat 34: 157–166. 10.1002/humu.22177 22887750 PMC3524353

[39] Goula AV, Berquist BR, Wilson DM 3rd, Wheeler VC, Trottier Y, et al (2009) Stoichiometry of base excision repair proteins correlates with increased somatic CAG instability in striatum over cerebellum in Huntington's disease transgenic mice. PLoS Genet 5: e1000749 10.1371/journal.pgen.1000749 19997493 PMC2778875

[40] Mason AG, Tome S, Simard JP, Libby RT, Bammler TK, et al (2013) Expression levels of DNA replication and repair genes predict regional somatic repeat instability in the brain but are not altered by polyglutamine disease protein expression or age. Human Molecular Genetics. 10.1093/hmg/ddt551 PMC3929096 24191263

[41] Sukhanova MV, Khodyreva SN, Lebedeva NA, Prasad R, Wilson SH, et al (2005) Human base excision repair enzymes apurinic/apyrimidinic endonuclease1 (APE1), DNA polymerase beta and poly(ADP-ribose) polymerase 1: interplay between strand-displacement DNA synthesis and proofreading exonuclease activity. Nucleic Acids Res 33: 1222–1229. 15731342 10.1093/nar/gki266 PMC549570

[42] Liu Y, Prasad R, Beard WA, Hou EW, Horton JK, et al (2009) Coordination between polymerase beta and FEN1 can modulate CAG repeat expansion. J Biol Chem 284: 28352–28366. 10.1074/jbc.M109.050286 19674974 PMC2788885

[43] Chan NL, Guo J, Zhang T, Mao G, Hou C, et al (2013) Coordinated processing of 3' slipped (CAG)n/(CTG)n hairpins by DNA polymerases beta and delta preferentially induces repeat expansions. J Biol Chem 288: 15015–15022. 10.1074/jbc.M113.464370 23585564 PMC3663522

[44] Garg P, Stith CM, Sabouri N, Johansson E, Burgers PM (2004) Idling by DNA polymerase delta maintains a ligatable nick during lagging-strand DNA replication. Genes Dev 18: 2764–2773. 15520275 10.1101/gad.1252304 PMC528896

[45] Mollersen L, Rowe AD, Larsen E, Rognes T, Klungland A (2010) Continuous and periodic expansion of CAG repeats in Huntington's disease R6/1 mice. PLoS Genet 6: e1001242 10.1371/journal.pgen.1001242 21170307 PMC3000365

[46] Lee JM, Pinto RM, Gillis T, St Claire JC, Wheeler VC (2011) Quantification of age-dependent somatic CAG repeat instability in Hdh CAG knock-in mice reveals different expansion dynamics in striatum and liver. PLoS One 6: e23647 10.1371/journal.pone.0023647 21897851 PMC3163641

[47] Evans AR, Limp-Foster M, Kelley MR (2000) Going APE over ref-1. Mutat Res 461: 83–108. 11018583 10.1016/s0921-8777(00)00046-x

[48] Lindahl T, Nyberg B (1972) Rate of depurination of native deoxyribonucleic acid. Biochemistry 11: 3610–3618. 4626532 10.1021/bi00769a018

[49] Nakamura J, Swenberg JA (1999) Endogenous apurinic/apyrimidinic sites in genomic DNA of mammalian tissues. Cancer Res 59: 2522–2526. 10363965

[50] Lokanga AR, Zhao X-N, Entezam A, Usdin K (2014) X inactivation plays a major role in the gender bias in somatic expansion in a mouse model of the Fragile X-related Disorders: implications for the mechanism of repeat expansion. Hum Mol Genet 23: 4985–4994. 10.1093/hmg/ddu213 24858908 PMC4140472

[51] Grasso M, Boon EM, Filipovic-Sadic S, van Bunderen PA, Gennaro E, et al (2014) A novel methylation PCR that offers standardized determination of FMR1 methylation and CGG repeat length without southern blot analysis. J Mol Diagn 16: 23–31. 10.1016/j.jmoldx.2013.09.004 24177047 PMC3873488

[52] Amouroux R, Campalans A, Epe B, Radicella JP (2010) Oxidative stress triggers the preferential assembly of base excision repair complexes on open chromatin regions. Nucleic Acids Res 38: 2878–2890. 10.1093/nar/gkp1247 20071746 PMC2875005

[53] Lan L, Nakajima S, Wei L, Sun L, Hsieh CL, et al (2014) Novel method for site-specific induction of oxidative DNA damage reveals differences in recruitment of repair proteins to heterochromatin and euchromatin. Nucleic Acids Res 42: 2330–2345. 10.1093/nar/gkt1233 24293652 PMC3936713

[54] Entezam A, Biacsi R, Orrison B, Saha T, Hoffman GE, et al (2007) Regional FMRP deficits and large repeat expansions into the full mutation range in a new Fragile X premutation mouse model. Gene 395: 125–134. 17442505 10.1016/j.gene.2007.02.026 PMC1950257

[55] Lavedan C, Grabczyk E, Usdin K, Nussbaum RL (1998) Long uninterrupted CGG repeats within the first exon of the human FMR1 gene are not intrinsically unstable in transgenic mice. Genomics 50: 229–240. 9653650 10.1006/geno.1998.5299

